# Arterial Spin‐Labeling MRI at the Cortical‐CSF Interface: A Novel Biomarker in Alzheimer Disease

**DOI:** 10.1002/acn3.70498

**Published:** 2026-08-07

**Authors:** Mona Asghariahmadabad, Pouya Metanat, Michael Thomas, Luke W. Bonham, Tyson Lam, Mehmet Kurt, Hong Nguyen, Tara Ellingson, Mauro Zucchelli, Stefano Casagranda, Quin Lu, Andreas M. Rauschecker, Leo Sugrue, Luis E. Savastano, Christopher P. Hess, Kamalini Ranasinghe, S. Andrew Josephson, Bruce L. Miller, Jennifer S. Yokoyama, Gil D. Rabinovici, Peter A. Ljubenkov, Lawren VandeVrede, Kambiz Nael

**Affiliations:** ^1^ Department of Radiology and Biomedical Imaging University of California, San Francisco San Francisco California USA; ^2^ Department of Mechanical Engineering University of Washington Seattle Washington USA; ^3^ Department of Neurology, Edward and Pearl Fein Memory and Aging Center, Weill Institute for Neurosciences University of California, San Francisco San Francisco California USA; ^4^ Department of Research and Innovation of Olea Medical La Ciotat France; ^5^ GE HealthCare San Francisco California USA; ^6^ Department of Neurological Surgery University of California, San Francisco San Francisco California USA

**Keywords:** Alzheimer disease, arterial spin‐labeling MRI, cortical‐CSF interface, perfusion biomarker

## Abstract

**Background/Objective:**

Arterial spin‐labeling (ASL) MRI can measure perfusion signal adjacent to CSF spaces and may provide information regarding CSF‐adjacent water transport physiology. We developed an automated pipeline to extract cortical‐CSF interface (IF) perfusion for comparison between Alzheimer disease (AD) and cognitively normal controls.

**Methods:**

In this retrospective study, participants with AD and cognitively normal controls with ASL and 3D T1‐weighted MRI were included. Parenchymal cerebral blood flow (CBF) was evaluated globally (whole brain, gray matter, white matter) and across 12 brain regions. IF‐perfusion values were quantified using an automated pipeline with tissue segmentation, registration, partial‐volume–aware masking, and arterial‐signal exclusion. Multivariable sensitivity analysis was performed, adjusting for age, sex, scanner vendor, and acquisition site. Predictive modeling was performed using LASSO‐regularized logistic regression with nested repeated stratified cross‐validation, and feature contributions were summarized using SHAP.

**Results:**

A total of 51 patients with AD, age (median: 73 years) and 53 cognitively normal subjects (median age: 69) were included. Global CBF did not differ between groups. However, IF‐perfusion values were significantly lower in AD (median 4.4, IQR 3.2–4.8) than in controls (median 5.2, IQR 4.5–6.3; *p* < 0.001). In multivariable analysis, AD remained independently associated with lower IF‐perfusion after adjustment for age, sex, scanner vendor, and acquisition site (β = −1.88, 95% CI: −3.52 to −0.25; *p* = 0.025). In LASSO‐regularized logistic regression model, only IF‐perfusion and cuneus‐CBF remained as significant contributing features for the prediction of AD probability with the highest contribution from IF. The final model showed mean ROC‐AUC 0.81 in the training and AUC of 0.78 in the testing dataset.

**Conclusion:**

ASL‐derived IF‐perfusion is reduced in AD and may represent a promising imaging marker of altered CSF‐adjacent water transport physiology. Further validation against established biomarkers of CSF dynamics and clearance pathways is warranted.

## 1 Introduction

Impaired cerebrospinal fluid (CSF) dynamics have been suggested to play a key role in the development of dementia including Alzheimer's disease (AD) [1]. The exchange of CSF within perivascular spaces (PVS) and interstitial fluid facilitates metabolic waste clearance from the brain through the glymphatic system. In patients with AD, disturbed CSF dynamics may contribute to impaired clearance of amyloid beta (Aβ) and tau, accelerating disease progression in both animal and human models [2, 3]. Although several imaging approaches have been proposed to assess the CSF dynamics such as diffusion tensor image analysis along the PVS (DTI‐ALPS) [4], blood oxygen level‐dependent CSF (BOLD‐CSF) coupling [5], and contrast‐enhancement along the perivascular spaces [6, 7], there is currently no consensus on the optimal imaging method.

Arterial spin‐labeled (ASL) estimated cerebral blood flow (CBF) has been used as a surrogate biomarker of vascular dysregulation which might precede detectable AD pathological changes, where regions with lower CBF have been linked to the presence of amyloid pathology in early disease [8, 9]. ASL signal within CSF has recently emerged as a promising approach for probing CSF dynamics [10, 11, 12]. Although conventional ASL quantification assumes a negligible CSF contribution to the perfusion signal, emerging evidence has shown that labeled water can exchange from the cerebral vasculature into CSF compartments, generating a detectable CSF‐associated ASL signal [12, 13, 14]. This observation suggests that ASL can provide complementary information beyond parenchymal CBF, enabling indirect assessment of water transport across vascular and perivascular pathways into CSF spaces.

However, reliable quantification of CSF‐adjacent ASL signal is technically nontrivial because measurements near the cortical surface are susceptible to partial‐volume mixing and macrovascular contamination [15]. Accordingly, recent methodological advances emphasize partial‐volume–aware sampling strategies and tissue‐fraction constraints to improve the specificity of cortical‐surface adjacent ASL measurements and better isolate CSF‐associated signal components [12].

Building on this emerging framework, we hypothesized that ASL‐derived perfusion signal at the cortical‐CSF interface can provide a practical surrogate of CSF‐water exchange adjacent to cortex and, by extension, represent a surrogate marker of altered CSF‐adjacent water transport physiology that may be associated with clearance processes in AD [16]. Therefore, in this study we aimed to develope an automated imaging pipeline to quantify the ASL‐perfusion measurements along the cortical‐CSF interface designed to reflect CSF‐adjacent physiology while minimizing partial‐volume and macrovascular confounding. We then performed a comparative analysis between patients with AD and age‐matched cognitively normal controls using both conventional parenchymal CBF and cortical‐CSF interface (IF) perfusion measurements.

## 2 Methods

### 2.1 Study Population

In this retrospective cohort study, we compared multi‐delay ASL derived perfusion measures between patients with AD and cognitively normal controls. The diagnosis of AD was established using clinical assessment and amyloid PET according to the 2024 National Institute on Aging‐Alzheimer's Association (NIA‐AA) criteria [17] who underwent pretreatment brain MRI at our institution from January to December of 2025. The control cohort was age‐matched at the group level and was primarily derived from ADNI‐4, supplemented by cognitively normal subjects from our institution with comparable MRI sequences.

Participants were included if they met the cohort definition (clinical AD for the patient group or cognitively normal status for controls) and had the required imaging inputs: (1) multi‐delay ASL and (2) a 3D T1‐weighted (3D‐T1W) anatomical image suitable for automated segmentation and registration. Participants were excluded if either ASL or 3D‐T1W was missing; or had nondiagnostic image quality (e.g., severe motion, incomplete brain coverage, or artifacts); or if key processing steps failed, including brain extraction, tissue segmentation/partial volume estimation, or T1‐to‐CBF registration, resulting in invalid masks or unreliable quantification. When multiple eligible scans were available for a subject, a single scan per subject was retained (earliest eligible pretreatment scan for AD; earliest eligible scan for controls).

For the AD cohort, we collected available baseline clinical and biomarker variables including *APOE* genotype, clinical stage at the time of imaging (mild cognitive impairment [MCI] vs. mild dementia), and baseline cognitive/functional scores (Mini‐Mental State Examination [MMSE], Clinical Dementia Rating Sum of Boxes [CDR‐SB], global CDR, and Functional Activities Questionnaire [FAQ]) when available.

This retrospective study was conducted under an approved institutional review board protocol (IRB No. 25‐43770). Written informed consent was waived due to the retrospective nature of the institutional cohort. Use of ADNI‐4 data complied with ADNI governance and data‐use policies. This study adheres to the Strengthening the Reporting of Observational Studies in Epidemiology (STROBE) reporting guideline.

### 2.2 Image Analysis

ASL studies were performed using a multi‐delay pseudo‐continuous ASL (pcASL) sequence on 3 T MRI scanners. ASL image acquisition parameters and postprocessing are summarized in the Supporting Information.

Imaging inputs for our processing workflow included a 3D‐T1W anatomical series and ASL‐derived CBF maps. All image processing was performed using standardized Python‐based workflow implemented with FSL tools and executed either in batch mode or through a graphical user interface (GUI). Identical processing settings were applied to the AD and control cohorts. FreeSurfer (v7.4.1) [18] (http://surfer.nmr.mgh.harvard.edu) was performed separately (outside the GUI/batch workflow) solely to generate anatomical segmentations used for regional CBF analyses.

**FIGURE 1:**
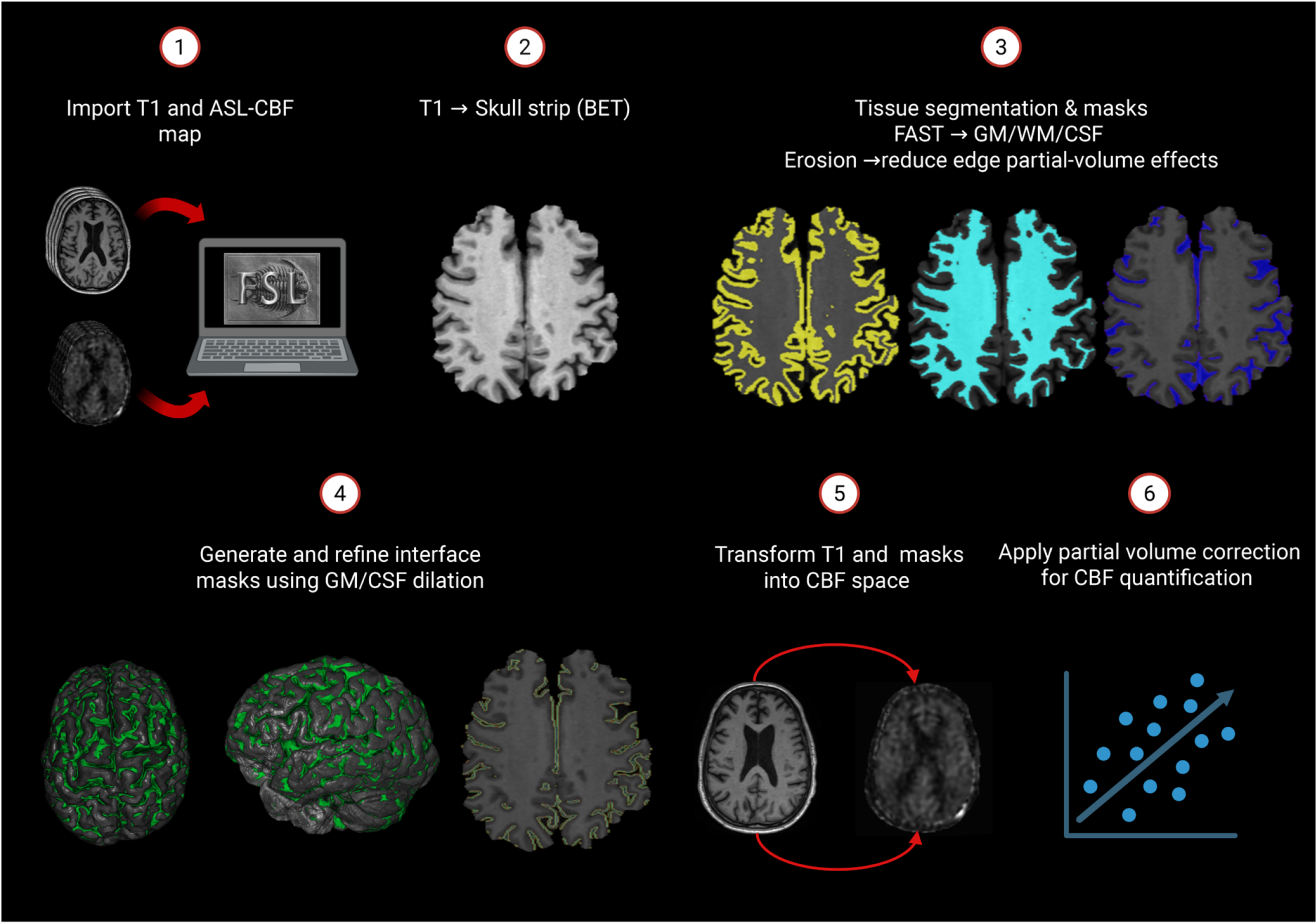
Overview of the ASL‐T1 processing workflow for cortical‐CSF interface (IF) and parenchymal CBF quantification. (1) T1‐weighted anatomical images and the corresponding ASL‐derived CBF maps are imported into the processing pipeline. (2) The T1 image is skull‐stripped to generate a brain‐only image and brain mask (FSL BET). (3) Tissue segmentation is performed on the brain‐extracted T1 image to estimate gray matter (GM), white matter (WM), and cerebrospinal fluid (CSF) partial‐volume maps (FSL FAST); tissue masks are refined with morphological erosions to reduce edge partial‐volume effects. (4) Cortical‐CSF interface (IF) masks are generated and refined using morphological dilation of the cortical GM mask and CSF‐based constraints to isolate voxels at the cortical boundary. (5) The T1 image and derived tissue/IF masks are transformed into ASL‐CBF space using rigid‐body alignment, enabling sampling of perfusion in the same space as the CBF map. (6) Partial‐volume correction is applied to support standardized parenchymal CBF quantification, and global/regional CBF metrics as well as IF‐perfusion extracted for downstream statistical analyses. (Created in https://BioRender.com).

Skull stripping of the 3D‐T1W series was performed to generate a brain‐only image and brain mask (FSL BET). Tissue segmentation (FSL FAST) was then performed on the brain‐extracted T1 image to estimate gray matter (GM), white matter (WM), and cerebrospinal fluid (CSF) partial volume fraction maps. Binary tissue masks were derived from these fraction maps using predefined thresholds, and morphological erosions were applied to reduce edge partial‐volume effects (GM: 2 erosions; WM: 1 erosion) [14]. To improve the specificity of interface measurements, additional “low partial‐volume” constraints were applied by excluding voxels with non‐negligible GM or WM fractions (threshold 0.10), thereby limiting interface quantification to CSF‐dominant voxels adjacent to cortex (Figure 1).

To align anatomical and perfusion data, the 3D T1‐weighted series were registered to the ASL‐CBF images using rigid/affine alignment implemented with FSL FLIRT and a mutual‐information cost function. The ASL‐CBF images were used as the fixed reference and T1‐weighted imaging as the moving images. The inverted transformation matrix was then applied to map T1‐derived tissue probability maps and anatomical masks into CBF space. Trilinear interpolation was used for probabilistic tissue‐fraction maps, whereas nearest‐neighbor interpolation was used for binary masks to preserve label integrity. All subsequent quantitative measurements and quality‐control analyses were performed in CBF space to avoid resampling of the perfusion maps.

### 2.3 Quantitative Analysis

#### 2.3.1 Cortical‐CSF Interface (IF) Perfusion

Interface masks were constructed in T1 space using morphological dilation of the GM mask. Two concentric interface rings were defined: IF1 was the first one‐voxel shell immediately external to cortical GM (dilation1 −GM), and IF2 was the subsequent shell (dilation2 −dilation1) (Figure S1). To restrict interface measurements to CSF‐dominant voxels and reduce tissue mixing, each ring was intersected with a strict CSF mask (CSF ≥ 0.90) and low partial‐volume constraints excluding voxels with non‐negligible GM or WM fraction (GM < 0.10 and WM < 0.10). The interface masks were then transformed into CBF space using the T1 to CBF transform and used to compute mean interface perfusion. To mitigate macrovascular contamination, voxels consistent with arterial signals were identified on the CBF map using an intensity threshold defined as the median perfusion value plus three times the median absolute deviation (MAD) within the candidate interface mask. Voxels exceeding this threshold were classified as probable arterial signal and excluded from the interface masks prior to quantification. The primary IF metric was defined as the mean perfusion value within the cleaned interface mask. IF1 was prespecified as the primary interface metric, whereas IF2 was used for agreement and sensitivity analyses [15]. See Table S2 for quality‐control assessment between cortical‐CSF interface masks.

#### 2.3.2 Partial‐Volume Correction and Regional Analysis

Partial‐volume correction (PVC) [12, 19] of perfusion was performed using a two‐compartment model that uses GM and WM tissue fractions in CBF space to estimate corrected GM and WM CBF maps. PVC outputs included corrected GM and WM perfusion maps used for subsequent parenchymal quantification.

Global CBF was computed within whole brain (brain mask), GM, WM, and IF. Mean regional CBF values were extracted from 12 FreeSurfer‐derived regional binary masks. Regional masks were transformed from T1 space into CBF space using the T1 to CBF transformation and nearest‐neighbor interpolation. Regional CBF was summarized as the mean values within each ROI.

### 2.4 Statistical Analysis

All statistical analyses were performed in Python 3.13.5 using statsmodels 0.14.6 and scikit‐learn 1.8.0. Continuous variables were summarized as median with interquartile range (IQR), and categorical variables as count (percentage). Baseline AD clinical and biomarker variables (*APOE* genotype, clinical stage, MMSE, CDR‐SB, global CDR, FAQ) were summarized descriptively for the AD cohort. Between‐group comparisons of perfusion metrics were performed using two‐sided Wilcoxon rank‐sum tests for continuous variables and Fisher exact tests for categorical variables, as appropriate. To address multiplicity, *p* values were adjusted using the Benjamini‐Hochberg false discovery rate (BH‐FDR) procedure separately across the group of global metrics (WB, GM, WM, IF1 and IF2) and the group of regional ROI comparisons. To assess the robustness of IF‐perfusion measurements to scanner and acquisition heterogeneity, an additional multivariable linear regression analysis was performed with IF‐perfusion as the dependent variable and diagnosis (AD vs. cognitively normal control), age, sex, scanner vendor, and acquisition site as independent variables. Robust HC3 standard errors (due to relatively small sample size) were used to account for potential heteroscedasticity. Regression coefficients, 95% confidence intervals (CI), and *p*‐values were reported.

Agreement between IF1 and IF2 was assessed using Pearson correlation and Bland–Altman analysis (mean bias and 95% limits of agreement) computed within each cohort.

To assess multivariable discrimination between AD and cognitively normal controls, a subject‐level Least Absolute Shrinkage and Selection Operator (LASSO) logistic regression model was developed to identify AD versus normal control, with all global and regional perfusion variables entered as candidate predictors. The dataset was divided into training and test sets using an 80/20 stratified split. To minimize data leakage, preprocessing was performed within the modeling pipeline using the training data only and included removal of variables with greater than 30% missingness, median imputation of remaining missing values, and z‐score standardization. The optimal degree of coefficient penalization was selected by grid search within a stratified 5‐fold cross‐validation procedure applied to the training data. Model performance during development was estimated using repeated nested stratified cross‐validation with 5 outer folds and 20 repeats, and discrimination was summarized by the mean ± standard deviation of area under the curve (AUC) and precision‐recall (PR)‐AUC across outer test folds. A final LASSO model was then fit on the training set using the selected hyperparameters and evaluated on the held out 20% test set. Final model coefficients and ROC analysis were derived from this fitted model. For model interpretability, SHapley Additive exPlanations (SHAP) values were computed using the final fitted model across the full dataset to provide a global summary of feature contributions; these SHAP results were considered descriptive. Statistical significance was defined as *p* < 0.05.

## 3 Results

### 3.1 Study Cohort and Demographics

**FIGURE 2:**
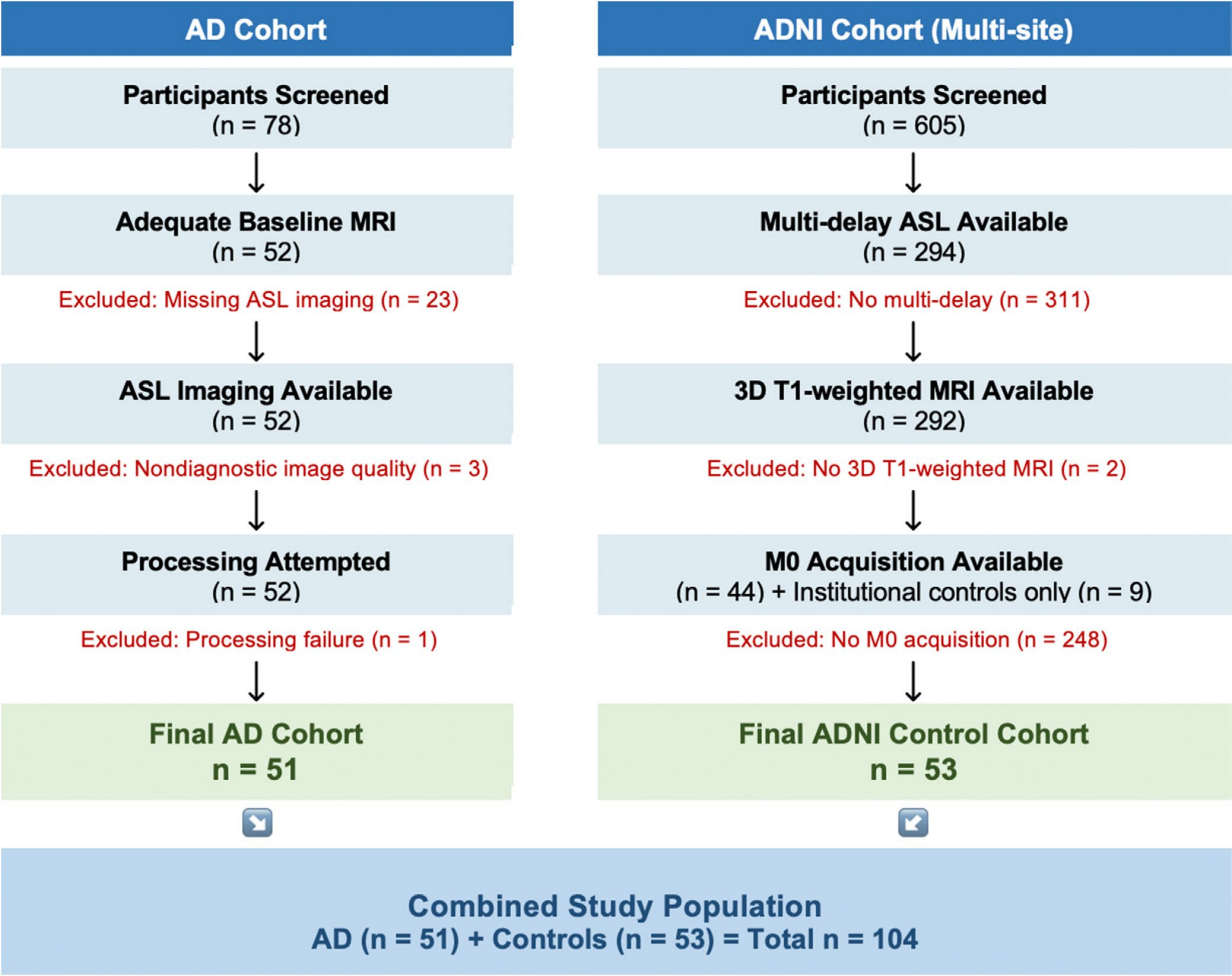
Study flow diagram. Participant selection and inclusion process for Alzheimer disease (AD) and cognitively normal control cohorts. The AD cohort was derived from patients with a clinical diagnosis of AD evaluated at UCSF. Cognitively normal controls were obtained from the Alzheimer's Disease Neuroimaging Initiative (ADNI) and supplemented with institutional controls. ADNI‐derived controls were required to have a multi‐delay arterial spin‐labeling (ASL) acquisition, a corresponding 3D T1‐weighted MRI, and an M0 acquisition suitable for quantitative processing. Subjects with missing imaging, nondiagnostic image quality, or failed image processing were excluded. The final study population consisted of 51 patients with AD and 53 cognitively normal controls.

A total of 51 patients with AD and 53 cognitively normal controls met inclusion criteria (Figure 2). Among the cognitively normal cohort, 44 subjects were included from ADNI data and 9 subjects were from our institution. The cohorts were comparable in age, with median (IQR) age of 73 (70–78) years in the AD group vs. 69 (65–75) years in controls. Sex distribution was similar between groups (female: 61.5% in AD vs. 61% in controls).

Scanner vendor and acquisition site distributions differed substantially between cohorts. Among patients with AD, 45/51 (88.2%) were scanned on GE systems and 6/51 (11.8%) on Siemens systems. All AD subjects were acquired at UCSF. Among cognitively normal controls, 44/53 (83.0%) were scanned on Siemens systems and 9/53 (17.0%) on GE systems. Most controls originated from ADNI (44/53, 83.0%), while 9/53 (17.0%) were acquired at UCSF Table S1.

In terms of clinical staging, our AD cohort consisted of 41 patients with mild cognitive impairment (MCI) and 10 patients with mild dementia. Baseline cognitive and functional measures (median, IQR) were 26, 25–28 for MMSE; 2, 1.5–3.5 for CDR‐SB; 0.5, 0.5–0.5 for global CDR; and 7, 3–11.5 for FAQ. *APOE* genotype breakdown included E3/E3 (53%), E3/E4 (35%) and E4/E4 (12%).

### 3.2 Comparative Analysis of Perfusion Values at Global Level

**FIGURE 3:**
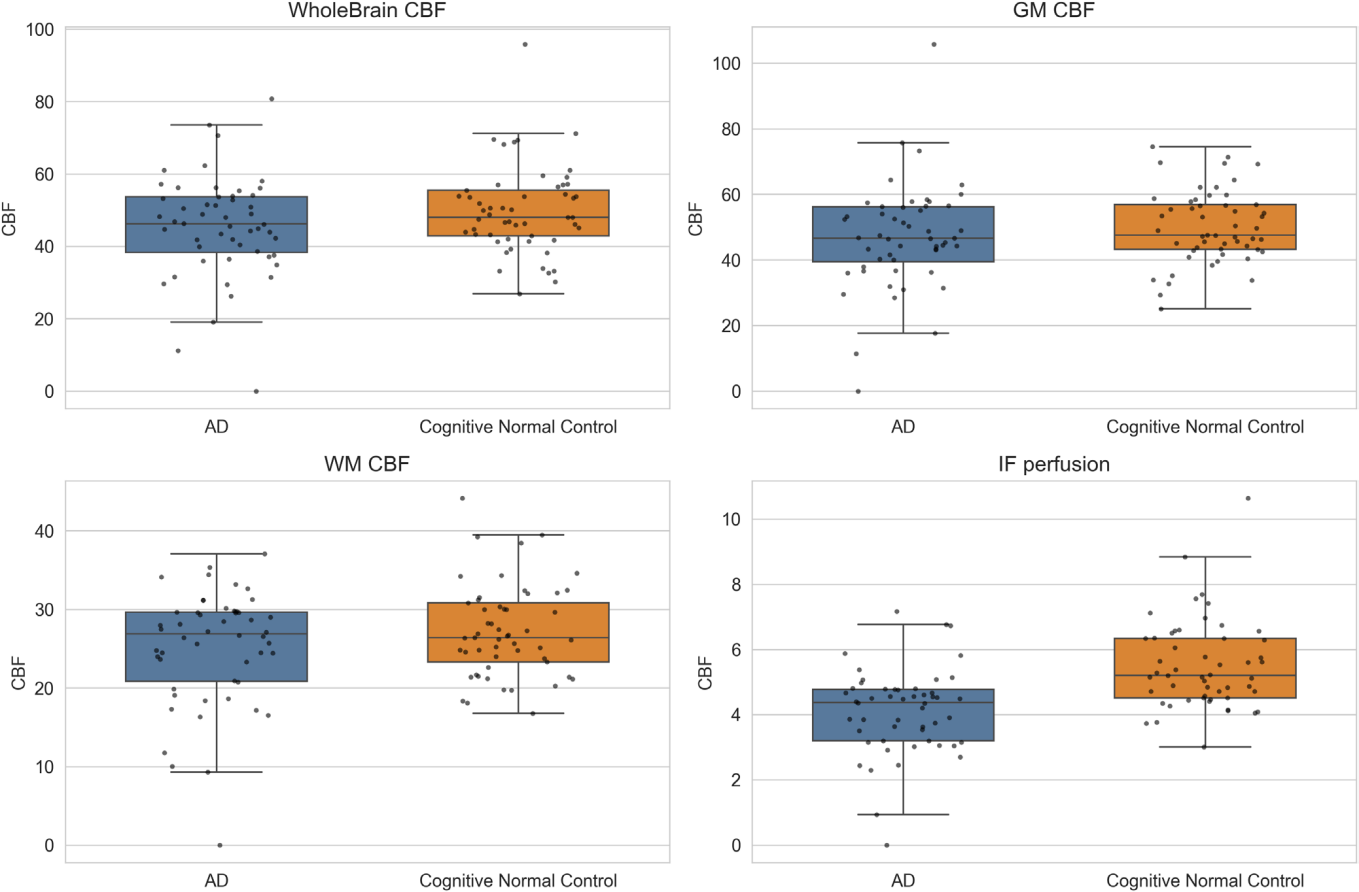
Group comparison of global and interface perfusion metrics in Alzheimer disease (AD) and cognitively normal controls. Box‐and‐whisker plots with overlaid individual subject values show ASL‐derived cerebral blood flow (CBF) for whole brain, gray matter (GM), white matter (WM), and cortical‐CSF interface (IF). Boxes indicate the interquartile range (IQR) with the median shown as the central line; whiskers denote the data range. Compared with cognitively normal controls, IF‐perfusion is lower in the AD cohort, whereas whole‐brain, GM, and WM CBF show greater overlap between groups.

Global parenchymal perfusion values measured for WB, WM, and GM were not significantly different between AD and cognitively normal cohort (Table 1). However, the cortical‐CSF interface (IF) perfusion values were significantly reduced in AD patients (median, IQR: 4.4, 3.2–4.8) in comparison with cognitively normal cohort (5.2, 4.5–6.3) (Table 1 and Figure 3). To quantify the magnitude of the observed group difference, the Hodges–Lehmann estimate of the median difference (AD minus cognitively normal controls) was calculated. IF‐perfusion was 0.892 units lower in AD than in controls (95% bootstrap confidence interval, −1.385 to −0.359), indicating a substantial reduction in interface perfusion associated with AD.

**TABLE 1:** Comparison of global perfusion and cortical‐CSF interface perfusion values among patients with AD (*n* = 51) and cognitively normal controls (*n* = 53).

Metric	AD, median [IQR]	Controls, median [IQR]	*p*	BH‐FDR *p*‐value*
Whole brain	46.2 [38.3–53.7]	48 [42.8–55.5]	0.19	0.32
Gray matter	46.6 [39.5–56.1]	47.6 [43.2–56.8]	0.24	0.32
White matter	26.9 [20.9–29.7]	26.4 [23.3–30.8]	0.37	0.37
Cortical‐CSF interface (IF)	4.4 [3.2–4.8]	5.2 [4.5–6.3]	< 0.001	< 0.001

*Note:* Perfusion values are in (mL/100 g/min).

* *p* values were adjusted using the Benjamini‐Hochberg false discovery rate (BH‐FDR) procedure within the variables of tests shown in the table.

For sensitivity analysis of interface definition, our results showed excellent agreement between IF1 and IF2 values in both cohorts. In the AD cohort, IF1 and IF2 values were nearly identical (*r* = 1.00) with negligible bias and tight limits of agreement. Similarly, in ADNI controls, agreement was near‐perfect (*r* = 1.00) with small bias (mean difference 0.01) and tight limits of agreement (0–0.01). Figure S2 shows Bland–Altman graphs comparing the IF1 and IF2 values across both cohorts.

To evaluate the impact of arterial‐signal exclusion on interface quantification, additional quality‐control analyses were performed. For IF1, the median number of voxels removed during arterial cleaning was 233 (IQR, 165–371) in the AD cohort and 1319 (IQR, 1049–1588) in cognitively normal controls, corresponding to median removal rates of 2.75% (IQR, 2.21%–4.05%) and 10.77% (IQR, 9.42%–12.18%), respectively. For IF2, the median numbers of removed voxels were 97 (IQR, 73–192) and 532 (IQR, 393–674), corresponding to median removal rates of 2.15% (IQR, 1.43%–4.15%) and 11.64% (IQR, 9.18%–15.14%), respectively (Table S2 and Figure S2).

To assess whether the observed AD‐control difference depended on arterial‐signal exclusion, a sensitivity analysis was performed using the uncleaned IF1 masks. The difference remained statistically significant before arterial cleaning (AD median 37.0 [IQR, 30.6–43.4] versus control median 42.8 [IQR, 37.9–48.9]; *p* = 0.0036), indicating that the reduction in IF‐perfusion observed in AD was not driven by the arterial‐signal exclusion procedure.

Moreover, additional quality‐control analyses were performed to characterize the interface masks. Voxel counts were quantified in both T1 and CBF spaces, and tissue‐fraction measurements were obtained within IF1 and IF2. Both interface masks demonstrated extremely high CSF purity with negligible gray‐ and white‐matter contamination. For IF1, median CSF fractions were 0.9997 in the AD cohort and 0.9985 in controls, whereas median GM fractions were 0.00029 and 0.00144, respectively. Median WM fractions were negligible in both groups. Similar findings were observed for IF2, where CSF fractions exceeded 0.99 in both cohorts with near‐zero GM and WM fractions (Table S2).

### 3.3 Comparative Analysis of Parenchymal Perfusion Values at Regional Level

Using FreeSurfer brain segmentation, CBF values were calculated in 12 brain segments including: Cuneus, Precuneus, Hippocampus, Parahippocampal gyrus, Amygdala, Insula, Caudate, Putamen, Globus pallidus, Cingulate gyrus, Superior frontal gyrus, and Middle frontal gyrus (Table 2). The CBF values were significantly (*p* < 0.001) lower in the cuneus in patients with AD (median, IQR: 16.6, 13.4–21.4) compared with controls (35.6, 23.0–45.0). This difference remained significant after BH‐FDR correction across all regions of interest (*p* = 0.001) (Table 2). The CBF values in the precuneus and hippocampus were also significantly lower in AD in comparison to normal cohort, however these differences did not remain significant after BH‐FDR correction (precuneus *p* = 0.06, hippocampus *p* = 0.09) (Table 2). CBF values among all other brain segments were not significantly different between patients with AD vs. cognitively normal cohort (Table 2).

**TABLE 2:** Regional comparison of parenchymal CBF values (mL/100 g/min) in patients with AD (*n* = 51) and cognitively normal cohort (*n* = 53).

Region	AD, median [IQR]	Controls, median [IQR]	*p*	BH‐FDR *p*‐value*
Cuneus	16.6 [13.4–21.4]	35.6 [23–45]	< 0.001	0.001
Precuneus	23 [20.3–29.3]	28.1 [22.6–39.1]	0.01	0.06
Hippocampus	28.7 [23.2–31.1]	32.3 [26.4–36.9]	0.02	0.09
Parahippocampal gyrus	26 [23.6–31.8]	30.7 [21.3–37.9]	0.24	0.48
Amygdala	30.9 [22.7–42.3]	27.1 [23.3–42]	0.79	0.85
Insula	24.5 [17.1–29.9]	26.6 [21.6–31.9]	0.10	0.24
Caudate	28 [21.9–33.4]	30.4 [22.5–37.7]	0.40	0.61
Putamen	30.6 [23.2–34.2]	30.4 [24.8–38.2]	0.35	0.59
Globus pallidus	31.5 [23.7–42]	31.4 [25.3–40.7]	0.74	0.85
Cingulate gyrus	28.4 [18.6–34.7]	25.4 [19.2–32.5]	0.69	0.85
Superior frontal gyrus	25.3 [16.3–29.1]	22.6 [18.9–28.1]	0.85	0.85
Middle Frontal gyrus	17.6 [12.3–22.8]	19.6 [15.7–24.9]	0.06	0.18

* *p* Values were adjusted using the Benjamini‐Hochberg false discovery rate (BH‐FDR) procedure within the variables of tests shown in the table.

To evaluate whether differences in ROI size could account for the observed cuneus perfusion findings, bilateral cuneus volumes were quantified in both T1 and CBF spaces. Median cuneus volumes were not significantly different between groups in either T1 space (AD: 5.72 mL [IQR, 5.05–6.80] versus controls: 6.43 mL [IQR, 5.43–7.11], *p* = 0.124) or CBF space (AD: 5.66 mL [IQR, 5.03–6.73] versus controls: 6.38 mL [IQR, 5.40–6.94], *p* = 0.115), suggesting that ROI size differences are unlikely to fully explain the observed reduction in cuneus perfusion.

### 3.4 Sensitivity Analysis for Scanner Vendor and Acquisition Site

After adjustment for age, sex, scanner vendor, and acquisition site, AD diagnosis remained independently associated with lower IF‐perfusion (β = −1.88, 95% CI: −3.52 to −0.25, *p* = 0.025). Scanner vendor was not independently associated with IF‐perfusion (GE versus Siemens: β = 0.51, 95% CI: −0.54 to 1.55, *p* = 0.34). These findings indicate that the observed reduction in IF‐perfusion persisted after accounting for acquisition‐related factors (Table 3).

**TABLE 3:** Multivariable sensitivity analysis adjusting for scanner vendor and acquisition site.

Variable	β coefficient	95% CI	*p*
AD diagnosis (vs cognitively normal control)	**−1.88**	**−3.52 to −0.25**	**0.025**
Age (years)	−0.01	−0.04 to 0.02	0.385
Female sex (vs male)	0.23	−0.27 to 0.72	0.371
GE scanner (vs Siemens)	0.51	−0.54 to 1.55	0.337
Brown University	−2.09	−3.58 to −0.60	0.006
Brown University (second site code)	−1.61	−2.92 to −0.30	0.017
Mayo Clinic Rochester	−0.60	−2.07 to 0.87	0.418
Rush University Medical Center	−1.94	−3.16 to −0.72	0.002
UAB Highlands	−0.55	−2.88 to 1.79	0.642
UCSF	−1.08	−3.29 to 1.13	0.333
University of Kentucky	−1.85	−3.02 to −0.68	0.002

*Note:* Bold values are the only significant variable in analysis.

### 3.5 Predictive Modeling

For model training, an 80/20 train‐test split was used. A LASSO‐regularized logistic regression model was trained with input including all global and regional perfusion values. Five‐fold cross validation was performed. In the final fitted LASSO model, only two features had non‐zero coefficients: IF (−1.8) and cuneus (−0.38) contributing to predicted probability of AD, with the lower values contributing to a higher predicted probability of AD. The final model using IF and cuneus perfusion values showed stable discrimination in nested repeated cross‐validation, with mean ROC‐AUC 0.81 ± 0.1 (95% CI, 0.79–0.83) and mean PR‐AUC 0.80 ± 0.11 (95% CI, 0.78–0.82) across 100 outer test folds. On evaluation in the independent held‐out test set, the final model achieved an AUC of 0.78 and a PR‐AUC of 0.83.

To evaluate the standalone diagnostic utility of IF‐perfusion, additional logistic regression models were developed using IF‐perfusion alone, cuneus perfusion alone, and the combination of IF‐perfusion and cuneus perfusion. The IF‐only model demonstrated good discrimination between AD and cognitively normal controls (AUC = 0.78, sensitivity = 71%, specificity = 66%). In comparison, the cuneus‐only model demonstrated lower discriminative performance (AUC = 0.61). Combining IF‐perfusion and cuneus perfusion yielded a modest improvement in performance (AUC = 0.81, sensitivity = 74%, specificity = 69%). In the IF‐only model, lower IF‐perfusion was significantly associated with AD (OR = 0.39, 95% CI: 0.25–0.60, *p* < 0.001). In the combined model, both IF‐perfusion (OR = 0.36, 95% CI: 0.22–0.60, *p* < 0.001) and cuneus perfusion (OR = 0.97, 95% CI: 0.94–0.99, *p* = 0.016) remained independently associated with AD.

**FIGURE 4:**
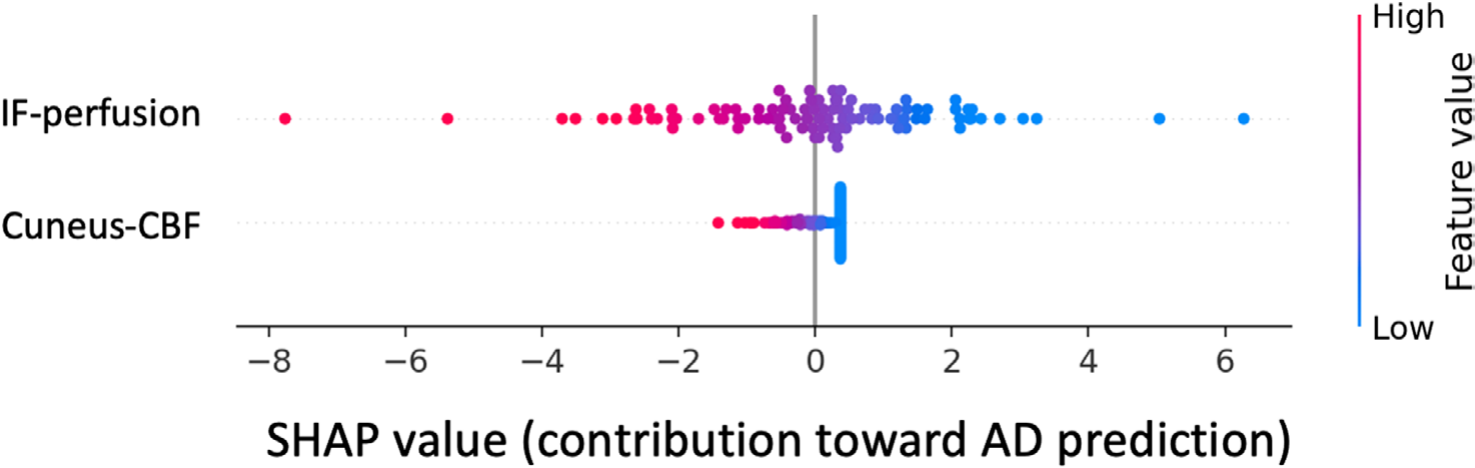
SHapley Additive exPlanations Beeswarm Plot of Feature Contributions to AD prediction. Each point represents an individual subject and corresponding SHAP contribution on the x‐axis (SHAP value) indicates how strongly that voxel's feature value pushes the model prediction toward AD (positive) or toward cognitively normal spectrum (negative). Color encodes the feature magnitude (low to high). The SHAP summary shows lower ASL perfusion values in IF and Cuneus (blue dots) are associated with higher model‐predicted probability of AD, whereas higher IF values (red dots) shift the predictions toward the cognitively normal cohort. IF is shown as the top and more important contributor for prediction.

Exploratory SHAP analyses were concordant and demonstrated that IF had the greatest contribution to model output (mean absolute SHAP 1.3) followed by cuneus (0.23) (Figure 4).

**FIGURE 5:**
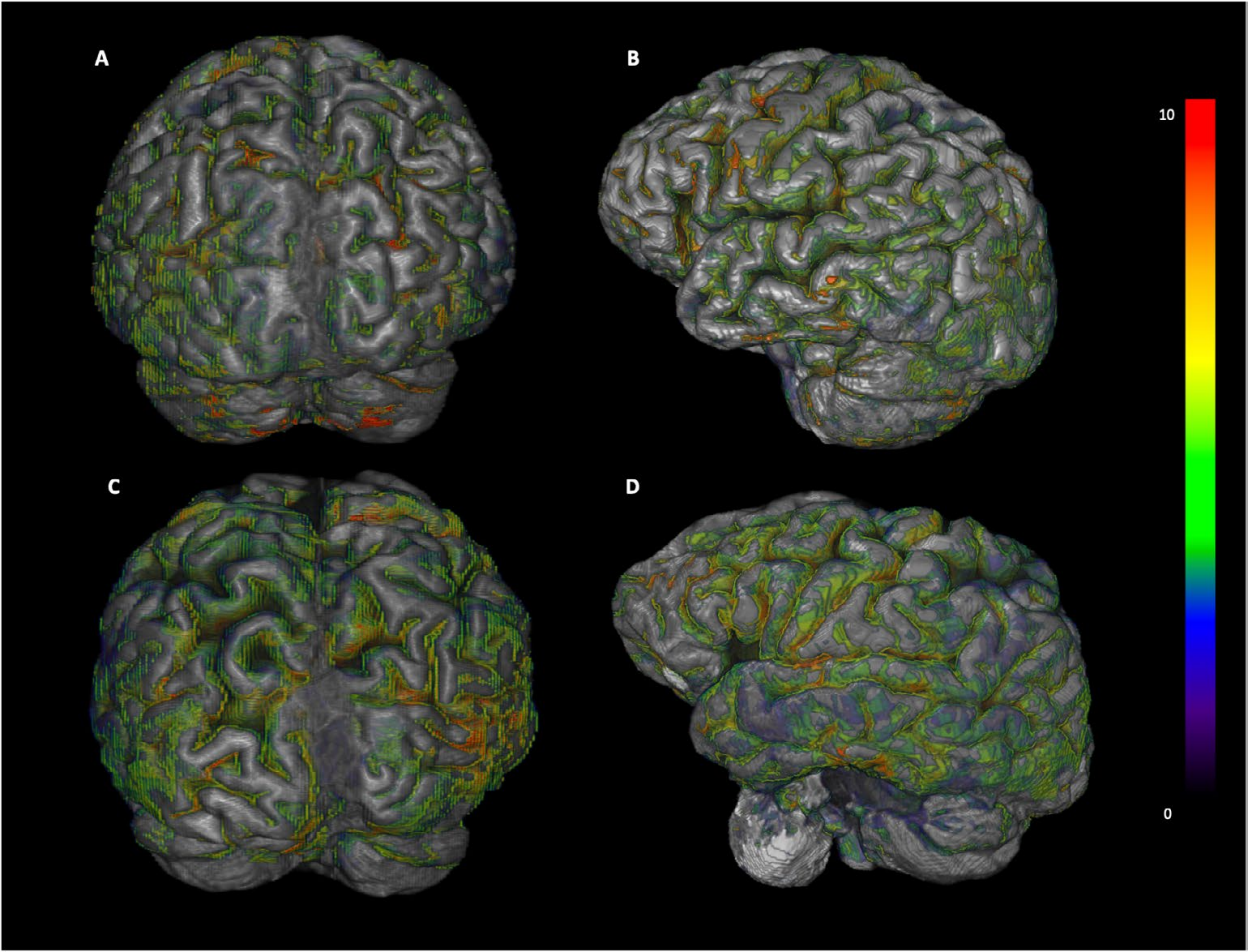
Representative 3D visualization of the cortical‐CSF interface (IF) perfusion overlaid on T1‐weighted anatomy. Surface renderings demonstrate the IF‐perfusion mask overlaid on the corresponding T1‐weighted images in a patient with Alzheimer disease (top row, panel A, B) and in a control participant with normal cognition (bottom row, panel C, D). Views are presented from posterior‐coronal and sagittal perspectives to illustrate the spatial distribution of the IF perfusion along the cortical‐CSF interface, with reduced values seen in patient with AD.

Correlation analysis between IF values and clinical cognitive or functional measures in AD cohort did not show significant correlation (MMSE, *r* = 0.008, *p* = 0.95; CDR‐SB, *r* = 0.013, *p* = 0.92; CDR‐Global, r = −0.04, *p* = 0.74; and FAQ, r = −0.02, *p* = 0.87). Similarly, the IF values did not differ significantly among patients with *APOE* subtypes of E3/E3, E3/E4 and E4/E4 (*p* = 0.56). Figure 5 shows comparison of topographical distribution of IF‐perfusion in a patient with AD vs. an age‐matched healthy control (Video S1 and S2).

## 4 Discussion

Our results showed that ASL‐derived perfusion along the IF is significantly reduced in patients with AD compared with age‐matched cognitively normal controls, despite the absence of a statistically significant difference in global whole‐brain, gray‐matter, or white‐matter CBF values. This dissociation suggests that IF captures a physiological feature that is not fully encapsulated by bulk parenchymal perfusion alone. The magnitude and consistency of the IF reduction across cohorts support the potential of this measure as an imaging surrogate of altered CSF‐tissue water transport in AD, aligning with growing evidence that impaired clearance pathways contribute to AD pathophysiology [20].

Prior work supports the biological plausibility of an ASL‐derived interface signal as a surrogate for impaired CSF‐water exchange and by extension, an imaging biomarker of CSF‐adjacent water transport physiology in AD [13, 16, 21]. ASL approaches with long inversion times have been used to quantify the clearance of labeled water from brain signal averages, with pilot data demonstrating slower clearance behavior in mild AD compared with controls [21]. In parallel, diffusion‐prepared, arterial spin labeling (DP‐ASL) techniques that estimate water exchange across the blood–brain barrier show that lower water exchange is associated with lower CSF Aβ42, consistent with reduced amyloid clearance capacity [22].

Similar to previous studies, we showed the feasibility of quantifying ASL derived cortical‐CSF interface‐focused perfusion and showed that it is significantly decreased in patients with AD compared with a cognitively normal age‐matched cohort. Our approach is unique in that we designed a dedicated and fully automated imaging pipeline to isolate the signal at the cortical‐CSF interface while reducing partial‐volume effects and macrovascular contamination using routine multi‐delay pcASL data. Additional quality‐control analyses demonstrated that both IF1 and IF2 masks were overwhelmingly CSF‐dominant with negligible gray‐ and white‐matter contamination, supporting the specificity of the interface measurements. Furthermore, the primary AD‐control difference remained statistically significant even before arterial‐signal exclusion, suggesting that the observed reduction in IF‐perfusion was not driven by the macrovascular cleaning procedure.

In our LASSO‐regularized logistic regression model, among all evaluated perfusion values at global and segmental level, decreased IF‐perfusion emerged as an independent and dominant contributing imaging biomarker in differentiating AD patients in comparison to normal controls. The only other independent contributing imaging variable was perfusion values in the cuneus; however, this contribution was modest in comparison to IF‐perfusion, as shown by our logistic regression and SHAP analysis. Additional logistic regression analyses demonstrated that IF‐perfusion alone achieved good discrimination between AD and cognitively normal controls (AUC = 0.78), whereas cuneus perfusion alone showed substantially lower performance (AUC = 0.61). Combining IF‐perfusion and cuneus perfusion yielded only a modest improvement in discrimination (AUC = 0.81). Our results together with existing data in the literature support a conceptual framework in which reduced cortical‐CSF interface perfusion values may reflect impaired CSF water transport which is mechanistically linked to waste clearance in aging and AD. Despite potential confounding from differences in scanner vendor and acquisition site, a multivariable sensitivity analysis adjusting for age, sex, scanner vendor, and site showed that Alzheimer's disease remained independently associated with reduced IF‐perfusion, indicating that the findings are unlikely to be explained by acquisition‐related variability (Table 3).

Alternative explanations for reduced IF‐perfusion should also be considered. First, ASL‐derived measurements may be influenced by vascular transit effects and delayed arrival of labeled water, which could alter the signal detected near the cortical surface independent of CSF‐water transport [15, 23]. Second, scanner vendor and acquisition differences may contribute to variability in perfusion measurements, particularly because the present study included both GE and Siemens datasets [15]. However, multivariable sensitivity analysis demonstrated that AD diagnosis remained independently associated with reduced IF‐perfusion after adjustment for age, sex, scanner vendor, and acquisition site. Third, cortical atrophy and enlargement of CSF spaces in AD could alter tissue composition at the cortical‐CSF boundary and potentially influence interface measurements. To mitigate this effect, our pipeline employed tissue‐fraction–based segmentation, strict CSF‐dominant masks, low‐partial‐volume constraints, and partial‐volume correction [12, 19]. Fourth, residual registration inaccuracies between anatomical and perfusion images could affect measurements at thin cortical boundaries; however, all masks were generated in high‐resolution T1 space and subsequently transformed into ASL space using rigid registration and nearest‐neighbor interpolation to preserve mask topology [15]. Finally, reduced IF‐perfusion may partially reflect regional perfusion abnormalities rather than exclusively CSF‐adjacent physiology [23, 24, 25, 26]. Nevertheless, global gray‐matter, white‐matter, and whole‐brain perfusion measurements did not differ significantly between groups, suggesting that IF‐perfusion is not simply a surrogate for global cerebral hypoperfusion.

Parenchymal perfusion assessment in our study showed lower CBF values within posterior cortical and medial temporal regions (notably the cuneus, precuneus, hippocampus) in patients with AD in comparison to cognitively normal cohort. This is concordant with existing literature [24, 25, 26, 27]. It is worth mentioning that among global perfusion measurements, IF‐perfusion was the only variable that was significantly reduced in the AD cohort while whole brain, white matter, and gray matter CBF values did not. This suggests that IF is not simply a proxy for global hypoperfusion in the AD cohort but provides complementary information beyond parenchymal CBF. The pathophysiology of reduced CBF in patients with AD is heterogeneous and has been attributed to multiple biological processes including neuronal activity, vascular dysfunction, and barrier/water‐exchange effects [24]. Independence of IF perfusion from global cerebral perfusion values in our study further supports that IF measures an additional physiological dimension constrained near CSF space and beyond conventional parenchymal perfusion alone.

If its potential is realized, IF‐perfusion measurement, which can be obtained from routinely acquired multi‐delay pcASL, can serve as a quantitative imaging biomarker for assessment of impaired CSF‐water transport in the AD population with potential diagnostic value for earlier detection of AD. In addition, as immunotherapies are being used for treatment of AD, IF‐perfusion could provide an opportunity for treatment response assessment, providing a window for longitudinal analysis of CSF‐water transport as a surrogate of CSF clearance in response to treatment.

This study has several limitations. First, the retrospective design introduces potential unknown bias. Second, we acknowledge heterogeneity in our ASL data by using a mix of GE and Siemens MRI studies and as the results heterogeneity of CBF maps since for GE studies we had to rely on vendor‐prespecified processing for CBF maps. This is however an inherent limitation of this type of study for real world experience similar to what is used for ADNI. Third, although our pipeline used rigorous tissue segmentation and partial‐volume‐constrained interface definition with macrovascular signal mitigation, IF‐perfusion remains an indirect surrogate of CSF‐tissue transport rather than a direct measurement of glymphatic flux; confirmatory studies that relate IF to established clearance biomarkers (e.g., CSF Aβ/tau, PET, or other glymphatic imaging methods) are needed. Fourth, multivariable modeling was exploratory and performed on a relatively small sample (51 ad vs. 53 normal cohort) and can be further validated with future prospective studies to improve generalizability. Fifth, we acknowledge substantial acquisition heterogeneity within the dataset. Most AD participants were acquired at UCSF using GE scanners, whereas most cognitively normal controls were derived from ADNI and acquired on Siemens platform. Although a multivariable sensitivity analysis demonstrated that reduced IF‐perfusion remained significantly associated with AD after adjustment for age, sex, scanner vendor, and acquisition site, residual confounding related to cohort source, scanner platform, and acquisition protocol cannot be completely excluded. Future prospective studies with harmonized imaging protocols can improve broader generalizability.

In conclusion, we introduced an automated imaging pipeline designed to reflect CSF‐adjacent perfusion while minimizing partial‐volume and macrovascular confounding. Our results showed that ASL‐derived IF‐perfusion is significantly reduced in AD in comparison to an age‐matched cognitively normal cohort. If validated in a larger prospective study, the described ASL‐derived IF measurement could be a promising, scalable imaging biomarker and surrogate for CSF water transport in patients with AD with potential diagnostic and therapeutic implications.

## Author Contributions

M.A., L.V., and K.N. conceived and designed the study. M.T., L.W.B., T.L., H.N., Q.L., A.M.R., L.S., L.E.S., C.P.H., K.R., S.A.J., B.L.M., J.S.Y., G.D.R., P.A.L., L.V., and K.N. contributed to data acquisition and/or participant recruitment. M.A., P.M., M.Z., S.C., and K.N. performed data processing and analyses. M.A., K.R., P.A.L., L.V., G.D.R., C.P.H., and K.N. interpreted the data. M.A., P.M., and K.N. drafted the manuscript. M.T., L.W.B., T.L., M.K., H.N., T.E., M.Z., S.C., Q.L., A.M.R., L.S., L.E.S., C.P.H., K.R., S.A.J., B.L.M., J.S.Y., G.D.R., P.A.L., L.V. and K.N. critically revised the manuscript for important intellectual content. K.N. supervised the overall project. All authors reviewed, approved the final manuscript, and agree to be accountable for all aspects of the work.

## Funding

The authors have nothing to report.

## Conflicts of Interest

Mona Asghariahmadabad, Pouya Metanat, Michael Thomas, Luke W. Bonham, Tyson Lam, Mehmet Kurt, Hong Nguyen, Tara Ellingson, Mauro Zucchelli, Stefano Casagranda, Quin Lu, Andreas M. Rauschecker, Leo Sugrue, Luis E. Savastano, Christopher P. Hess, Kamalini Ranasinghe, S. Andrew Josephson, Bruce L. Miller, Gil D. Rabinovici, Peter A. Ljubenkov, Lawren VandeVrede, and Consultant to Olea Medical for Kambiz Nael declare no conflicts of interest related to this work. Jennifer S. Yokoyama serves on the scientific advisory board for the Epstein Family Alzheimer's Research Collaboration, the Charleston Conference on Alzheimer's Disease, and Taudia Inc., and is the editor‐in‐chief of *npj Dementia*.

## Supporting information


**Figure S1:** Representative cortical‐CSF interface masks. IF1 (blue) and IF2 (yellow) masks are overlaid on a T1‐weighted anatomical image. Right side of the figure demonstrates the whole‐brain view, and left side shows a magnified view of the cortical‐CSF boundary. IF1 and IF2 were generated as adjacent concentric shells surrounding cortical gray matter using morphological dilation and CSF‐dominant tissue constraints. The masks are mutually exclusive by design and were used to assess the robustness of interface quantification.
**Figure S2:** Bland–Altman agreement between IF1 and IF2 interface perfusion measurements metrics in AD and cognitively normal controls. Bland–Altman plots show the difference between the two cortical‐CSF interface metrics (IF1−IF2) versus their mean for the AD cohort and the cognitively normal control cohort. Each point represents one subject. The blue dashed line indicates the mean difference (bias), and the red dashed lines indicate the 95% limits of agreement (bias ±1.96 SD). Overall, the plots demonstrate the level of agreement between IF1 and IF2 within each cohort and highlight any systematic bias or variability across the range of interface perfusion values.
**Table S1:** Scanner vendor and Site distribution.
**Table S2:** Quality‐control metrics for cortical‐CSF interface masks.
**Table S3:** Performance and coefficients of simplified logistic regression models.

**Video S1:** Representative cortical‐CSF interface (IF) perfusion in a patient with Alzheimer disease. Three‐dimensional rendering of the cortical‐CSF interface (IF) perfusion overlaid on 3D T1‐weighted anatomical images. The video demonstrates the spatial distribution of perfusion across the cortical‐CSF interface (IF). Note reduced IF perfusion across the cortical–CSF interface in this patient with Alzheimer disease compared with the cognitively normal subject in Video 2.

**Video S2:** Representative cortical‐CSF interface (IF) perfusion in a cognitively normal control participant. Three‐dimensional rendering of the cortical‐CSF interface (IF) perfusion overlaid on the 3D T1‐weighted anatomical images. The video demonstrates the spatial distribution of perfusion across the cortical‐CSF interface (IF). Note higher IF perfusion across the cortical–CSF interface in this cognitively normal subject compared with the patient with Alzheimer disease in Video 1.

## Data Availability

The data that support the findings of this study are available from the corresponding author upon reasonable request.
