# Supplementary material for: Arterial Spin‐Labeling MRI at the Cortical‐CSF Interface: A Novel Biomarker in Alzheimer Disease
Source: Ann Clin Transl Neurol. 2026 Aug 7:10.1002/acn3.70498. Online ahead of print. doi: 10.1002/acn3.70498 (PMC13448560; doi:10.1002/acn3.70498)
Supplement: Supplementary file 1 [file ACN3-9999-0-s003.docx]

**ASL acquisition**

We used time-encoded multi-delay pseudo continuous ASL (pcASL) sequence to mitigate the underestimation of CBF in regions affected by long transit times.

For GE scanners, image acquisition was performed on SIGNA Premier 3T scanners using the following sequence parameters: TR = 7304 ms, TE = 22.5 ms, FOV = 256 mm, spatial resolution = 1.8 × 1.8 × 4 mm³, BW = 125 kHz, label duration₁₋₇ = 600 ms, seven post-label delay₁₋₇ = 700, 1271, 1843, 2414, 2986, 3557, 4129 ms, NEX = 2.

For Siemens scanners, image acquisition was performed on MAGNETOM Vida 3T scanners using the following sequence parameters: TR = 4800 ms, TE = 24.1 ms, FOV = 240 mm, spatial resolution = 1.8 × 1.8 × 4 mm³, BW = 223.2 kHz, label duration₁₋₇ = 800 ms, a range of post-label delay₁₋_9_ = 800, 1150, 1500, 1850, 2200, 2550, 2900, 3250, 3500 ms, NEX = 2.

**CBF Processing:**

A pcASL signal model derived from the general kinetic model^1^ was used to generate the theoretical ASL signal at each PLD. Arterial transit time (ATT) was measured using a weighted delay method^2^ . A perfusion-weighted curve was computed at each TI, and a two-compartment model (tissue and arterial)^3^ was applied. The CBF maps from each delay were then combined through a weighted average to generate transit-delay-corrected CBF.

For ASL studies performed on GE scanners, where M0 images are not accessible due to proprietary constraints, CBF maps were generated using the vendor-provided inline tool on the MR scanners. For studies acquired on Siemens scanners, ASL image analysis was performed using Olea Sphere 3.0 (Olea Medical, La Ciotat, France) to generate quantitative CBF maps utilizing the available proton-density reference image (M0) for standardized quantification.

**Figure S1. Representative cortical-CSF interface masks.** IF1 (blue) and IF2 (yellow) masks are overlaid on a T1-weighted anatomical image. Right side of the figure demonstrates the whole-brain view, and left side shows a magnified view of the cortical-CSF boundary. IF1 and IF2 were generated as adjacent concentric shells surrounding cortical gray matter using morphological dilation and CSF-dominant tissue constraints. The masks are mutually exclusive by design and were used to assess the robustness of interface quantification.

**Figure S2. Bland-Altman agreement between IF1 and IF2 interface perfusion measurements metrics in AD and cognitively normal controls.**
Bland-Altman plots show the difference between the two cortical-CSF interface metrics (IF1 − IF2) versus their mean for the AD cohort and the cognitively normal control cohort. Each point represents one subject. The blue dashed line indicates the mean difference (bias), and the red dashed lines indicate the 95% limits of agreement (bias ± 1.96×SD). Overall, the plots demonstrate the level of agreement between IF1 and IF2 within each cohort and highlight any systematic bias or variability across the range of interface perfusion values.

**Table S1: Scanner vendor and Site distribution**

| **Variable** | **AD (n=51)** | **Controls (n=53)** |
| --- | --- | --- |
| **Scanner Vendor** |  |  |
| GE | 45 (88.2%) | 9 (17.0%) |
| Siemens | 6 (11.8%) | 44 (83.0%) |
| **Acquisition Site** |  |  |
| UCSF | 51 (100%) | 9 (17.0%) |
| ADNI | 0 (0.0%) | 44 (83.0%) |

**Table S2. Quality-control metrics for cortical-CSF interface masks**

| **Metric** | **AD Median [IQR]** | **Controls Median [IQR]** |
| --- | --- | --- |
| IF1 voxels in CBF | 8006 [7292-8847] | 11578 [10050-13545] |
| IF1 voxels removed | 233 [165-371] | 1319 [1049-1588] |
| IF1 voxels removed (%) | 2.75 [2.21-4.05] | 10.77 [9.42-12.18] |
| IF2 voxels in CBF | 4540 [3789-5483] | 4043 [3597-5402] |
| IF2 voxels removed | 97 [73-192] | 532 [393-674] |
| IF2 voxels removed (%) | 2.15 [1.43-4.15] | 11.64 [9.18-15.14] |
| IF1 mean CSF fraction | 0.9997 [0.9996-0.9998] | 0.9985 [0.9983-0.9987] |
| IF1 mean GM fraction | 0.00029 [0.00022-0.00043] | 0.00144 [0.00121-0.00165] |
| IF1 mean WM fraction | <0.0001 | <0.0001 |
| IF2 mean CSF fraction | 0.999999 [0.999998-1] | 0.99994 [0.99990-0.99999] |
| IF2 mean GM fraction | ~0 | ~0 |
| IF2 mean WM fraction | ~0 | ~0 |

**Table S3. Performance and coefficients of simplified logistic regression models**

| **Model** | **Predictor(s)** | **AUC** | **Sensitivity** | **Specificity** | **Odds Ratio** | **95% CI** | **p-value** |
| --- | --- | --- | --- | --- | --- | --- | --- |
| IF-only logistic regression | IF | 0.776 | 0.712 | 0.660 | 0.39 | 0.25-0.60 | <0.001 |
| Cuneus-only logistic regression | Cuneus | 0.611 | 0.600 | 0.490 | 0.97 | 0.94-0.99 | 0.006 |
| IF + Cuneus logistic regression | IF | 0.810 | 0.740 | 0.686 | 0.36 | 0.22-0.60 | <0.001 |
|  | Cuneus |  |  |  | 0.97 | 0.94-0.99 | 0.016 |

**References:**

1.	Buxton RB, Frank LR, Wong EC, Siewert B, Warach S, Edelman RR. A general kinetic model for quantitative perfusion imaging with arterial spin labeling. *Magn Reson Med*. Sep 1998;40(3):383-96. doi:10.1002/mrm.1910400308

2.	Dai W, Robson PM, Shankaranarayanan A, Alsop DC. Reduced resolution transit delay prescan for quantitative continuous arterial spin labeling perfusion imaging. *Magn Reson Med*. May 2012;67(5):1252-65. doi:10.1002/mrm.23103

3.	Woods JG, Achten E, Asllani I, et al. Recommendations for quantitative cerebral perfusion MRI using multi-timepoint arterial spin labeling: Acquisition, quantification, and clinical applications. *Magn Reson Med*. Aug 2024;92(2):469-495. doi:10.1002/mrm.30091
